# Integrating transcriptome and chemical analyses to reveal the anti-Alzheimer’s disease components in *Verbena officinalis* Linn

**DOI:** 10.3389/fpls.2022.955075

**Published:** 2022-08-04

**Authors:** Shuhuan Peng, Fangyi Li, Kuo Yu, Fengshu Zhou, Heshui Yu, Hui Liu, Jialiang Guo, Guoqiang Li, Chunhua Wang, Xiaohui Yan, Zheng Li

**Affiliations:** ^1^School of Medicine, Foshan University, Foshan, China; ^2^College of Pharmaceutical Engineering of Traditional Chinese Medicine, Tianjin University of Traditional Chinese Medicine, Tianjin, China; ^3^School of Food Science and Engineering, Foshan University, Foshan, China

**Keywords:** Alzheimer’s disease, *Verbena officinalis*, transcriptome, content determination, tissue expression difference, biosynthetic pathway

## Abstract

*Verbena officinalis* Linn. is a kind of traditional Chinese medicine, which has a long history of application and shows good effects on neuroprotection. Therefore, we consider that *V. officinalis* may be a potential drug for treating Alzheimer’s disease (AD). First, ultra-performance liquid chromatography-mass spectrometry (UPLC-MS) pointed out that the main chemical components in *V. officinalis* were iridoid glycosides, phenylethanoid glycosides, and flavonoids. These compounds were used for molecular docking and the results showed that these compounds had good anti-AD activity. To explore the biosynthetic pathway of anti-AD components in *V. officinalis*, UPLC and ultraviolet (UV) spectrophotometry were used for contents determination and the result was leaf > stem > root. At the same time, 92,867 unigenes were annotated in *V. officinalis* transcriptome; 206, 229, 115 related unigenes were, respectively, annotated in iridoid glycoside, phenylethanoid glycoside, and flavonoid pathway, of which 61, 73, and 35 were differential expression genes. The components had relatively high expression in leaves, which was consistent with the quantitative results. In addition, the tissue distribution particularity of verbenalin may be related to the branching of pathways. Meanwhile transcription factors VoWRKY6 and VoWRKY7 may be involved in the regulation of iridoid glycoside biosynthesis. Further, VoWRKY3, VoWRKY9, and VoWRKY12 may be related to flavonoid biosynthesis. The above research is helpful to explore the biosynthetic pathway of anti-AD components and the regulation mechanism of active components and to further explore the anti-AD effect of *V. officinalis*.

## Introduction

Alzheimer’s disease (AD) is a common neurodegenerative disease, which mostly occurs among elderly people. Its clinical symptoms are mainly expressed as hypomnesia, thinking slow, and language and learning disability. As humans get older, the risk of disease increases. Nowadays, with the trend of global aging, the threat of AD to human beings has increased. At present, the pathogenesis of AD is not clear. Researchers have put forward many related hypotheses, including amyloid hypothesis, tau hyperphosphorylation hypothesis, neuroinflammation hypothesis, mitochondrial dysfunction hypothesis, cholinergic hypothesis, and so on (Holmes, 2013; Lane et al., 2018; DeTure and Dickson, 2019). The drugs for clinical treatment of AD include acetylcholinesterase (AChE) inhibition galantamine, lisdimene, tacrine, donepezil, and N-methyl-D-aspartate receptor antagonist memantine (Ghezzi et al., 2013). Although these drugs can effectively improve the symptoms of AD, they cannot fundamentally treat AD.

*Verbena officinalis* Linn. (Verbenaceae), is a popular herb in folk medicine for the treatment of skin disease, edema, and dysmenorrhea. Pharmacological studies have shown that it has many pharmacological effects such as anti-tumor, antibacterial, antiviral, anti-early pregnancy, anti-inflakangmatory, and antioxidantion (Kubica et al., 2020). Notably, *V. officinalis* also shows a good neuroprotective effect. Lai et al. (2006) treated neurons *in vitro* with aqueous extract, which could reduce both destructions of neurites and neuronal apoptosis. The plant aqueous extract may play a role by inhibiting the activation of caspase-2 and caspase-3 by nerve cells. Tan and Wang (2011) prepared AD mice by giving D-galactose. After administration of *V. officinalis* decoction, they found that the number of first dark avoidance errors after 24 h significantly reduced, indicating that *V. officinalis* decoction improved its directional learning and memory ability. Outside, *V. officinalis’* good anti-inflammatory effects may also be a potential factor in the treatment of AD (Calvo et al., 1998; Speroni et al., 2007; Grawish et al., 2016).

There are many chemical components in *V. officinalis*, and the material basis of its anti-AD activity is not clear. Because the main components of the plant are iridoid glycosides, flavonoids, and phenylethanoid glycosides, they are considered active ingredients for anti-AD. Iridoid glycosides are characteristic components of this plant and a large number of studies have shown that the biosynthetic pathway of iridoid glycosides is the same as that of other terpenoids. Geranyl diphosphate is produced through the mevalonate (MVA) or 2-C-methyl-D-erythritol-4-phosphate (MEP) pathway, and iridoid glycosides are generated through the downstream synthesis pathway (Krithika et al., 2015; Ahmad et al., 2018). Many studies have used RNA sequencing (RNA-Seq) technology to explore the biosynthetic pathway of medicinal plants which is rich in iridoid glycoside such as *Lamium barbatum* (Li et al., 2010), *Gardenia jasminoides* (Pan et al., 2021), and *Rehmannia glutinosa* (Sun et al., 2012). The flavonoid biosynthetic pathway is relatively clear, mainly involving the phenylpropane pathway, which has been clarified in *Arabidopsis thaliana* and other plants (Saito et al., 2013; Lou et al., 2021). In addition, the flavonoid biosynthetic pathway is also associated with secondary metabolites such as flavonol, flavanone, dihydroflavonol, and anthocyanin (Liu et al., 2021). Acteoside is a typical phenylethanoid glycoside compound. In the biosynthesis of acteoside, caffeic acid is produced by the phenylalanine pathway and 3-hydroxyloside is generated by the dopamine pathway or tyramine pathway. Bai et al. (2014) isolated a glycosyltransferase from *Rhodiola rosea*, UGT73B6, which can catalyze tyrosol to generate salidroside. And salidroside is a precursor of 3-hydroxyloside biosynthesis in *Escherichia coli*. However, the cross pathway between downstream acyl transfer and glycosyl modification is not clear and needs to be further explored.

To explore the anti-AD active compounds of *V. officinalis*, we first analyzed the chemical components by ultra-performance liquid chromatography-mass spectrometry (UPLC-MS) to investigate the potential therapeutic effects and possible mechanisms for AD. Iridoid glycosides, flavonoids, and phenylethanoid glycosides were docked with AD target protein by molecular docking technology. Then, we quantitatively analyzed the active components to determine the tissue difference in component content. Meanwhile, the RNA-Seq technology was used for *V. officinalis*. With the help of gene expression difference analysis and quantitative analysis of tissue differences, we speculated the enzymes related to the biosynthesis of active components and the biosynthetic pathway involving anti-AD active components, to provide clues for the further study of the anti-AD activity of *V. officinalis*.

## Materials and methods

### Preparation of plant materials

**FIGURE 1:**
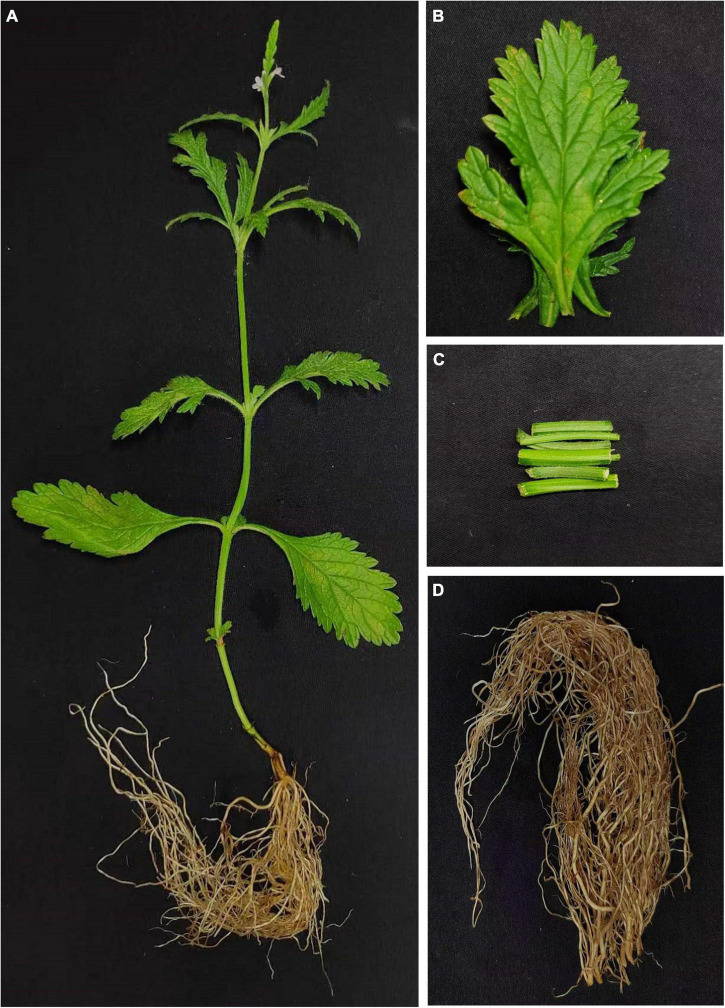
Plant and tissues of *Verbena officinalis*. **(A)** Whole *V. officinalis*. plant. **(B)** Leaves. **(C)** Stems. **(D)** Roots.

We herborized *V. officinalis* seedlings (7 May 2021) from Wangshan, Huangtan, Wencheng, Wenzhou, Zhejiang, China (27°44’10.23” N, 119°58’2.12” E). They were cultured at the Tianjin University of Traditional Chinese Medicine, in July 2021 and identified by Dr. Wang Chunhua. Plant samples are stored in the Pharmaceutical Engineering College of Tianjin University of Traditional Chinese Medicine. We selected normal growth and disease-free plants, and washed the tissue with pure water, sterile water, and RNA-free water. Quickly cut the plant leaf, stem, and root tissues into small pieces (three biological replicates for each tissue, from three plants cultured under the same conditions, a total of nine samples). Then, quickly froze them with liquid nitrogen, and transferred them to the refrigerator at –80°C for standby (Figure 1). After fresh plants were picked, the leaf, stem, root, and total plant tissue dried naturally, and we ground them into powder for sample.

### Ultra-performance liquid chromatography-mass spectrometry

The Waters ACQUITY UPLC™ System (Waters, Milford, MA, United States) combined with Q Exactive™ Plus (Q Exactive Plus–Orbitrap MS., Thermo Fisher Scientific., Waltham, MA, United States) and Xcalibur 4.1 software (Thermo Fisher Scientific., Waltham, MA, United States). Acetonitrile and methanol used in the experiment are chromatographic pure reagents (Thermo Fisher Scientific., Waltham, MA, United States) and pure water from Mill-Q system (Millipore., Illkirch-Graffenstaden, France). The chromatographic column is Waters Acquity UPLC^®^ BEH C_18_ column (100 mm × 2.1 mm, 1.7 μm, Waters, Milford, MA, United States), column temperature was 25°C, the mobile phases were acetonitrile(A) and water (B) with a flow rate of 0.2 ml/min, and the injection volume was 1 μl. The gradient program was 0–1 min, 5% A; 1–35 min, 5–95% A; 35–40 min, 95%A. Q Exactive MS source parameters include heated electrospray spray ionization source (HESI), positive and negative mode detection, 100-1,500 Da in full MS scan mode, a 3.5 kV spray voltage, a 320°C capillary temperature, a 350°C auxiliary gas heater temperature, with a sheath gas flow rate of 40 arb, and the auxiliary gas flow rate at 15 arb. The resolution of the full MS was 70,000 and the resolution of the dd-MS2 was 17,500.

The total plant powders (0.1 g) were added to 5 ml of 80% (v/v) methanol for ultrasonic extraction (power 250 W, frequency 40 kHz), and the extraction time was 2 h. The solution was filtered after cooling, and 80% (v/v) methanol was added to the solution a total 5 ml. The test solution was centrifuged at 14,000 r/min at 4°C for 15 min, and the supernatant was taken for 0.22 μm filter membrane for UPLC-MS.

### Molecule docking

The 3D structure of small molecule was downloaded from PubChem.^1^ NLRP3 (6NPY), BACE1 (6UWP), AChE (6CQZ), and GSK-3β (1J1C) were downloaded from the PDB database.^2^ PyMol 2.5.0 software was used for the water removal and ligand removal of target proteins, and the hydrogenation was completed with Autodock Tools 1.5.6. Finally, Autodock vina software was used for the docking of small molecules and target proteins.

### Analysis of tissue contents difference

The Agilent Series 1290 UPLC (Agilent Technologies., Santa Clara, CA, United States) was used to determine the contents of verbenalin and acteoside. Standard references are verbenalin (Saizhiwei Technology Co., Tianjin, China, content ≥ 98%, batch number: wkq21100922) and acteoside (Saizhiwei Technology Co., Tianjin, China, content ≥ 98%, batch number: wkq22031108). Acetonitrile is chromatographic pure (Thermo Fisher, Waltham, MA, United States), pure water is taken from Mill-Q (Millipore., Illkirch-Graffenstaden, France), and methanol is analytically pure (Concord Technology., Tianjin, China). The chromatographic column is Waters Acquity UPLC^®^ BEH C_18_ column (100 mm × 2.1 mm, 1.7 μm, Waters, Milford, MA, United States), column temperature was 30°C, the mobile phases were acetonitrile (A) and water (B) with a flow rate of 0.2 ml/min, detective wave was 240 nm and 334 nm, and the injection volume was 1 μl. The gradient program was 0–1 min, 5% A; 1–3 min, 5–23% A; 3–13 min, 23–95% A. The standard curve equation with 240 nm of verbenalin was *Y* = 7517.9*X* + 44.166, *R*^2^ = 0.9993, the linear range was 0.032625 ∼ 0.58 mg/ml. And the standard curve equation with 334 nm of acteoside was *Y* = 3814.3*X* – 23.406, *R*^2^ = 0.9996, the linear range was 0.08775 ∼ 1.56 mg/ml.

The sample powders (0.1 g) were added to 10 ml of 80% (v/v) methanol for ultrasonic extraction (power 250 W, frequency 40 kHz), and the extraction time was 2 h. The solution was filtered after cooling, and 80% (v/v) methanol was added to solution a total 10 ml. The test solution was centrifuged at 10,000 r/min for 10 min, and the supernatant was taken for 0.22 μm filter membrane for UPLC analysis. Each sample was measured three times and the experiment was repeated three times for parallel to calculate the content of chemical components in each tissue.

Otherwise, we used Agilent Cary 8454 UV-Vis (Agilent Technologies., Santa Clara, CA, United States) to determine the contents of total iridoid glycosides, phenylethanoid glycosides, and flavonoids in *V. officinalis*. verbenalin (Saizhiwei Technology Co., Tianjin, China., content ≥ 98%., batch number: wkq21100922), as the main component of *V. officinalis* was used as the quantitative analysis of total iridoid glycosides. The standard curve equation with 240 nm of total iridoid glycosides was *Y* = 26.489*X* + 0.0071, *R*^2^ = 0.9995, the linear range was 4.716 ∼ 29.868 μg/ml. For the quantitative determination of phenylethanoside, acteoside (Saizhiwei Technology Co., Tianjin, China., content ≥ 98%., batch number: wkq22031108) was used as standard. At 334 nm, the standard curve equation was *Y* = 26.354*X* –0.0047, *R*^2^ = 0.9999, the linear range was 4.496 ∼ 29.224 μg/ml. For the content determination of total flavonoids, apigenin (Saizhiwei Technology Co., Tianjin, China., content ≥ 98%., batch number: wkq21072811) was selected as the standard control, colored with 1% (v/v) triethylamine, and analyzed quantitatively at 383 nm. The standard curve equation was *Y* = 121.5*X* –0.002, *R*^2^ = 0.9998, the linear range was 0.944 ∼ 6.136 μg/ml. The sample powders (0.1 g) were added to 20 ml of 80% (v/v) methanol for ultrasonic extraction (power 250 W, frequency 40 kHz), and the extraction time was 2 h. The solution was filtered after cooling, and 80% (v/v) methanol was added to solution a total 100 ml. Each sample was measured three times and the experiment was repeated three times in parallel to calculate the content of chemical components in each tissue.

### RNA extraction, cDNA library construction, and sequencing

The leaf, stem, and root tissues of *V. officinalis* (3 copies each for biological replicate) were used to extract the total RNA from the cryopreserved plant tissues by using the Plant Total RNA Purification Kit (Tiangen biotech., Guangzhou, China) according to the instructions. The RNA quality was evaluated by using Agilent 2100 Bioanalyzer (Agilent Technologies., Santa Clara, CA, United States), and the degradation degree of RNA was detected by agarose gel electrophoresis. Based on the structural characteristics of most eukaryotic mRNAs with PolyA tail, the mRNA with PolyA tail was enriched by oligo (dT) magnetic beads, fragmented and reverse transcribed by ultrasound, and then repaired at the end. A total of 9 cDNA libraries were obtained by PCR amplification with linker, index, and sequencing primers. Each cDNA library was sequenced on Illumina Novaseq 6000 platform with paired end. The sequencing was done by Novogene (Novogene Co., Ltd., Beijing, China).

### *De novo* assembly and unigene annotation

The original data were transformed into sequence data through base identification and presented in the fastq format. FastQC was used to ensure the quality and reliability of data analysis, the reads with adapter, reads containing, and low-quality reads were moved to obtain clean data. The clean reads data were assembled *de novo* into transcripts using Trinity (Grabherr et al., 2011). Based on Trinity splicing, Corset (Davidson and Oshlack, 2014) software was used for hierarchical clustering. After removing redundancy, take the longest transcript in each transcript cluster as a non-repetitive sequence gene.

In order to obtain the functional information of genes, the program blast2go (Götz et al., 2008) was used to compare unigenes with NR (NCBI non-redundant protein sequences), NT (NCBI nucleoside sequences), Pfam (Protein family), KOG/COG (Clusters of Orthologous Groups of proteins/euKaryotic Ortholog Groups) and Swiss prot (A manually annotated and reviewed protein sequence database), KEGG (Kyoto Encyclopedia of Genes and Genomes), and GO (Gene Ontology) database.

### Differential expression analysis

Taking the transcriptome by Trinity as the reference sequence, Bowtie2 program (Langmead and Salzberg, 2012) was used to map the clean reads into the transcript assembly, and RSEM software (Li and Dewey, 2011) for the FPKM value (expected number of fragments per kilobase of transcript sequence per million base pairs sequence) to measure the quantitative analysis of gene expression level. Differential expression genes (DEGs) were screened (screening standard was | log_2_(FoldChange)| > 1 and *p* < 0.005) by DESeq2 (Love et al., 2014).

We performed GO function enrichment and KEGG pathway enrichment analysis on the DEGs. The biosynthetic pathways of iridoid glycosides, phenylethanoid glycosides, and flavonoids in references, combined with the results of database function annotation, and relevant genes were identified. The value of FPKM was used to measure the level of expression. It is used to predict the biosynthetic pathway of related compounds. ChemDraw 20.0 and Origin 2021 were used to draw the heat map of the biosynthesis pathway and gene expression, respectively.

The iTAK software (Zheng et al., 2016) was used to predict plant transcription factors. In addition, we downloaded the sequence of WRKY transcription factors (TFs) in various plants from NCBI, and used MAGA7 (Kumar et al., 2016) software to analyze the developmental tree of WRKY in *A. thaliana, Dendrobium officinale, Catharanthus roseus, Artemisia annua, Oryza sativa, R. glutinosa*, and *V. officinalis*. Sequence alignment was performed with MUSCLE (Edgar, 2004). The sequence was cut with TBools (Chen C. et al., 2020) and the phylogenetic tree was constructed with Neighbor-Joining with 1,000 bootstrap replicates. The iTOL^3^ tools were used to beautify the phylogenetic tree.

## Results

### Ultra-performance liquid chromatography-mass spectrometry

In this study, UPLC-MS was used to analyze and identify the chemical components of *V. officinalis* extract under positive and negative ion modes, respectively. There were responses in both positive and negative ion modes. A total of 16 compounds were identified, including 8 compounds in the positive ion mode and 8 compounds in the negative ion mode (Supplementary Figure 1). We scanned the medicinal materials in the positive and negative modes (Table 1). The results showed that the main chemical components of *V. officinalis* were iridoid glycosides, phenylethanoid glycosides, and flavonoids, among which iridoid glycosides mainly included verbenalin, hastatoside, gentiopicroside, aucubin, 3,4-dihydroverbenalin, and swertiamarine. Phenylethanoid glycosides include acetoside, isoacteoside, 2′-acetylacetoside, jionoside D, and cistanoside F. Flavonoids mainly include acacetin-7-*O*-rutinoside, apigenin-7-*O*-glucoside, glucosyl-6-pedalitin, 4’-hydroxyl wogonoside, and acacetin.

**TABLE 1:** Result of UPLC-MS in *Verbena officinalis*.

Type	Compound	Molecular weight	Measured mass (*m/z*)	Ion mode	Retention time	MS/MS fragment
Iridoid glycosides	Aucubin	346.3	345.10	[M-H]^–^	1.11	183.09, 179.06, 167.11, 149.09
	Swertiamarine	374.3	373.11	[M-H]^–^	1.21	419.12, 179.06, 149.04, 141.02
	Hastatoside	404.4	427.12	[M + Na]^+^	6.44	243.09, 225.08, 207.06, 193.05
	Gentiopicroside	356.3	357.12	[M + H]^+^	6.77	195.07, 177.05
	Verbenalin	388.4	389.14	[M + H]^+^	7.03	357.12, 227.09, 195.06
	3,4-Dihydroverbenalin	390.4	391.16	[M + H]^+^	7.87	229.11, 197.08
Phenylethanol glycosides	2’-Acetylacetoside	666.2	665.21	[M-H]^–^	1.08	503.16, 179.05
	Acetoside	624.6	623.20	[M-H]^–^	9.34	461.20, 315.11, 161.02
	Cistanoside F	488.4	487.14	[M-H]^–^	10.04	179.03, 161.02, 135.04
	Jionoside D	638.6	637.21	[M-H]^–^	10.53	461.07, 193.05, 175.07, 135.04
	Isoacteoside	624.6	623.20	[M-H]^–^	10.99	461.17, 161.02
Flavonoids	Apigenin-7-*O*-glucoside	432.3	433.10	[M + H]^+^	7.56	445.08, 269.05
	Glucosyl-6-pedalitin	478.1	477.09	[M-H]^–^	8.15	300.06, 271.91, 166.02
	4’-Hydroxyl wogonoside	462.1	463.28	[M + H]^+^	22.49	477.27, 301.20, 269.21
	Acacetin	284.0	283.04	[M-H]^–^	24.14	241.03, 159.03, 153.09, 135.04, 115.07, 111.08
	Acacetin-7-*O*-rutinoside	592.5	593.28	[M + H]^+^	25.32	447.32, 285.20, 270.21, 242.28

The UPLC-MS results showed that the main compounds in *V. officinalis* were iridoid glycosides, phenylethanoid glycosides, and flavonoids. Literature research shows that iridoid glycosides are characteristic components of *V. officinalis*, and verbenalin has a high content (Council of Europe, 2020). As a representative of phenylethanoid glycosides, acetoside also has a high content in *V. officinalis* (Deepak and Handa, 2000). The above two components were identified in UPLC-MS, and flavonoids were also identified in the experiment. Therefore, we consider that the above three compounds are the main anti-AD active components of *V. officinalis*. These components are the main direction to study the anti-AD effect of *V. officinalis*.

### Molecular docking

To investigate the potential of chemical components for the treatment of AD, we chose target proteins for docking with compounds in *V. officinalis*. Chronic inflammation is suspected to be associated with the occurrence of AD. The activation of NLRP3 leads to the release of cytokines, which plays a key role in the pathogenesis of AD (Shao et al., 2015; Chen D. B. et al., 2020). The deposition of Aβ produces neurotoxicity, which maybe leads to the occurrence of AD. Beta-site APP Cleaving Enzyme1 (BACE1) is the rate-limiting enzyme for Aβ, and its activity reduction can prevent Aβ deposition (Moussa-Pacha et al., 2020). Elenbecesta was used as a control to measure the effect of the compounds on BACE1 (Das et al., 2021). Acetylcholinesterase (AChE) can degrade acetylcholine. When the AChE activity increases, the concentration of acetylcholine decreases, which may lead to the loss of acetylcholine at cholinergic synapses, and lead to cognitive and memory impairment. Donepezil is a clinically applied ChE inhibitor and is used as the reference drug in this experiment (Ambure et al., 2014). Hyperphosphorylation of tau protein will destroy the structure and physiological function of neurons. The expression level of GSK-3β is related to the phosphorylation of tau protein. In the experiment, TWS119 was used as a positive control drug (Eskandarzadeh et al., 2021). The NLRP3, BACE1, AChE, and GSK-3β are closely related to Alzheimer’s disease. Supplementary Figure 2 shows the relationship between these target proteins and AD (Loera-Valencia et al., 2019).

**FIGURE 2:**
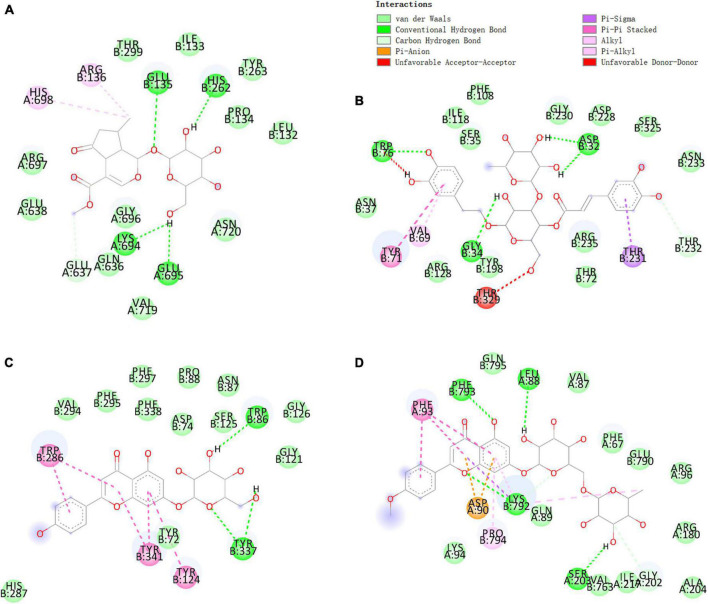
Molecular docking of some compounds in *Verbena officinalis*. with AD targets. **(A)** verbenalin docking with NLRP3. **(B)** Acetoside docking with BACE1. **(C)** Apigenin-7-*O*-glucoside with AChE. **(D)** Acacetin-7-*O*-rutinoside docking with GSK-3β. The figure on the upper left explains that different colors represent different types of compounds that bind with amino acids.

Verbenalin, hastatoside, aucubin, swertiamarine, acetoside, isoacteoside, jionoside D, cistanoside F, acacetin-7-*O*-rutinoside, apigenin-7-*O*-glucoside, glucosyl-6-pedalitin, and acacetin were used to molecular docking (Table 2). Verbenalin (Figure 2A), swertiamarin, and jionoside D have a good binding effect with NLRP3, which may play a role in the treatment of AD by reducing inflammation. Phenylethanoid glycosides including acetoside (Figure 2B) and isoacteoside have good docking effects with NLRP3 and BACE1, which may reduce the inflammatory response and inhibit Aβ deposition. Apigenin-7-*O*-glucoside (Figure 2C) and glucosyl-6-pedalitin were higher than positive drugs in molecular docking with NLRP3, BACE1, and AChE. Acacetin-7-*O*-rutinoside (Figure 2D) has a good binding effect on all four protein targets, which is better than that of apigenin-7-*O*-glucoside, glucosyl-6-pedalitin. Acacetin also has multi-target effects.

**TABLE 2:** Docking results of *Verbena officinalis*. compounds with AD target protein molecules.

Type	Compounds	NLRP3	BACE1	AChE	GSK-3β
		Binding affinity (kcal/mol)	Binding affinity (kcal/mol)	Binding affinity (kcal/mol)	Binding affinity (kcal/mol)
Positive control	Itanapracedyang	–7.9	–	–	–
	Elenbecesta	–	–8.2	–	–
	Donepezil	–	–	–9.8	–
	TWS119	–	–	–	–9.8
Iridoid glycosides	Verbenalin	**–8.4**	–7.9	–7.9	–8.8
	Aucubin	–7.5	–7.1	–7.4	–7.1
	Swertiamarine	**–8.1**	–7.4	–7.4	–8.4
Phenylethanol Glycosides	Acetoside	**–8.8**	**–8.8**	–8.5	–8.0
	Isoacteoside	**–9.0**	**–8.5**	–9.1	–9.4
	Jionoside D	**–8.9**	–8.2	–8.7	–8.7
	Cistanoside F	–7.7	–7.3	–7.3	–7.6
Flavonoids	Acacetin-7-*O*-tinoside	**–10.6**	**–9.5**	**–9.6**	**–10.2**
	Apigenin-7-*O*-glucoside	**–9.8**	**–9.1**	**–10.0**	–9.1
	Glucosyl-6-pedalitin	**–9.6**	**–8.4**	**9.9**	–9.3
	Acacetin	**–8.3**	**–8.3**	9.3	–8.5

The words in red are those with a binding energy greater than the positive drug.

### *De novo* assembly and unigene annotation

There were the sequencing data of 9 cDNA libraries (3 biological replicates in each tissue) and obtained 62.55 GB clean data. The clean data of each sample reached more than 6 GB. The overall sequencing error rate of each sample was 0.03% (the error rate of all libraries was 0.03%), Q20 base ratio more than 97.21%, Q30 base ratio more than 92.3%, indicating that the data were qualified and meet the analysis requirements (Supplementary Table 1).

**FIGURE 3:**
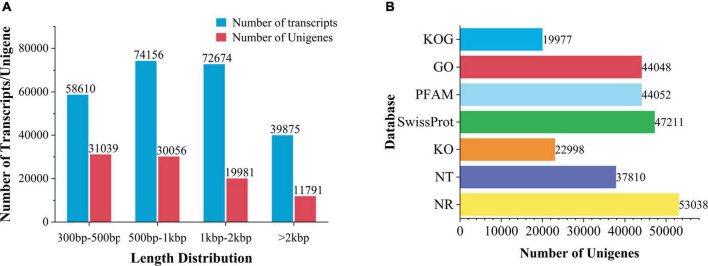
Transcription splicing and database annotation. **(A)** Transcripts and Unigenes length distribution. **(B)** Number of gene annotations in the database.

High-quality sequences were spliced by Trinity to obtain 2,45,315 transcripts, with an average length of 1,208 bp. After removing redundant and clustering analysis, 92,867 unigenes were obtained, with an average length of 10,44bp (Supplementary Table 2 and Figure 3A).

Unigenes were compared to 7 databases for gene function annotation. Unigenes annotated in NR for 57.11% of the total base number, NT 40.71%, Pfam 47.43%, go 47.43%, Ko 24.76%, Swissprot 50.83%, and KOG 21.51% (Figure 3B). In addition, 71.68% of unigenes in *V. officinalis* were annotated in at least one database and 7.78% of genes were annotated in seven databases.

**FIGURE 4:**
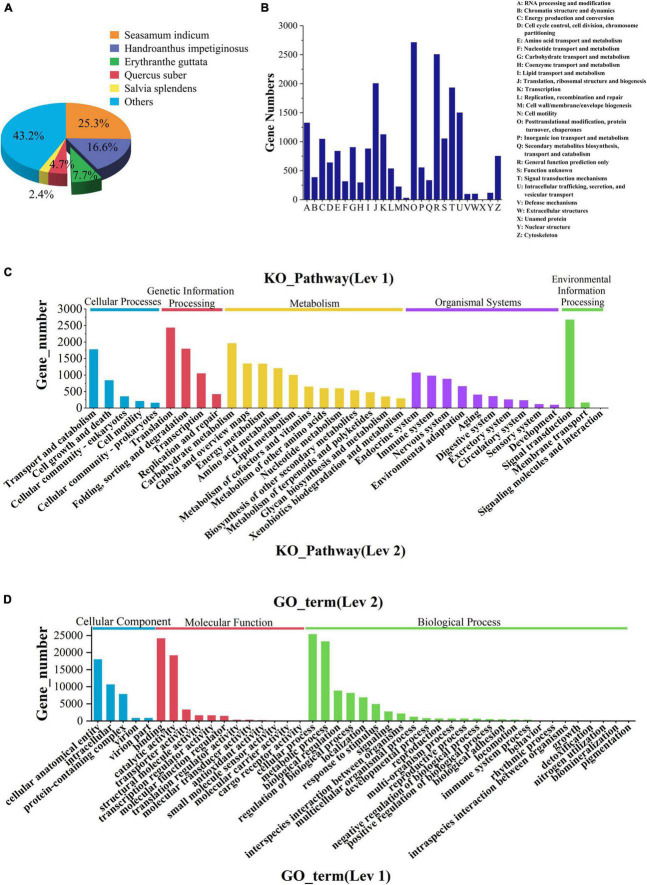
Database annotation classification. **(A)** Species distribution of transcriptomic unigenes against NR database. **(B)** KOG classification. **(C)** KEGG pathway classification. **(D)** GO classification.

In the comparison of the NR database, it was found that the species with a high matching degree with *V. officinalis* similar sequence are *Sesamum indicum* (accounting for 25.3%), followed by *Handroanthus impetiginosus* (accounting for 16.6%), *Erythranthe guttata* (accounting for 7.7%), *Quercus suber* (accounting for 4.7%), and the others less than 2.5% (Figure 4A).

The function prediction and classification statistics of unigenes can be carried out through the KOG database. A total of 19,977 unigenes were annotated in the KOG database and were classified according to 26 groups (Figure 4B and Supplementary Table 3). The five highest numbers are Post-translational modification (2,720 unigenes), General function prediction only (2,514 unigenes), Translation, ribosomal structure and biogenesis (2,013 unigenes), Signal translation mechanisms (1,940 unigenes), and Internal trafficking and secret and vesicular transport (1,508 unigenes).

There were 4 KEGG metabolic pathways in *V. officinalis* transcriptome, including 34 branches and 302 metabolic pathways (Figure 4C and Supplementary Table 4). Among them, the top five pathways are Ribosome (1,043 unigenes), Carbon metabolism (832 unigenes), Spliteosome (792 unigenes), Protein processing in endoplastic reticulum (722 unigenes), and Biosynthesis of amino acids (647 unigenes).

GO functions are divided into biological process, cellular component, and molecular function. In the annotation results, unigenes of the three types of functions are involved, and 43 branches are annotated in the second level (Figure 4D and Supplementary Table 5). In the biological process category, it mainly involves cellular process (25,366 unigenes), metallic process (23,237 unigenes), and biological regulation (8,832 unigenes). In the cell group classification, it mainly includes cellular analytical entity (18,034 unigenes), intracellular (10,686 unigenes), and protein-containing complex (7,873 unigenes). Among the molecular functional categories, they are mainly concentrated in binding (24,152 unigenes), catalytic activity (19,166 unigenes), and transporter activity (3,289 unigenes).

### Differential expression analysis

**FIGURE 5:**
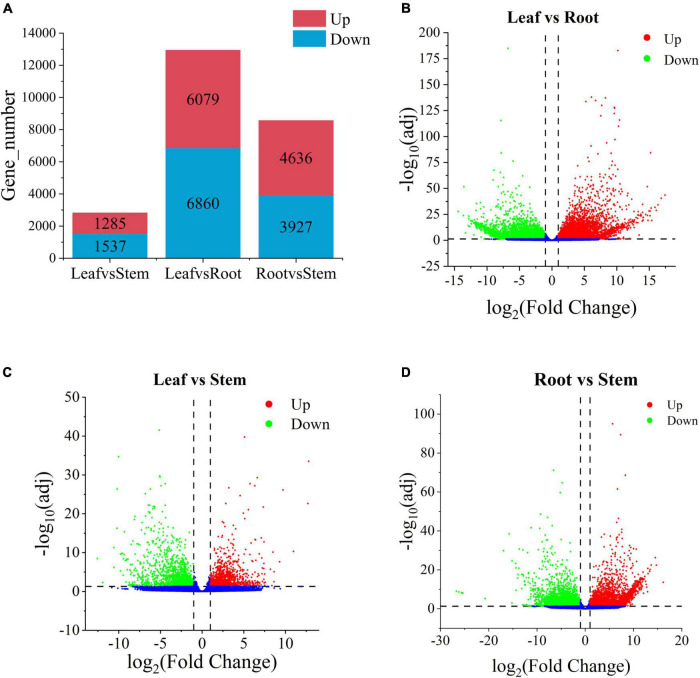
Histogram of the number of differential genes in each tissue of and the volcanoplots of differential genes in *Verbena officinalis*. **(A)** Histogram of differential genes. **(B)** Volcanoplot of Leaf vs. Root. **(C)** Volcanoplot of Leaf vs. Stem. **(D)** Volcanoplot of Root vs. Stem DEGs. The screening criteria for DEGs are *p* < 0.05 and | log_2_FoldChange| > 1 by DESeq2.

To explore the metabolic differences of *V. officinalis* in different tissues, the samples were analyzed by differential expression analysis. A total of 92,867 unigenes were annotated in *V. officinalis* transcriptome, including 15,715 DEGs (Figure 5A). Under the condition of *p* < 0.05 and | log2foldchange | > 1, the differential genes between the tissues were screened.

There are 12,939 DEGs in the leaf vs. root group (Figure 5B and Supplementary Table 6), with the largest number, and the up-regulated genes account for 47.0%. Therefore, it is speculated that there may be great differences in contents of secondary metabolites in leaf vs. root. Leaf vs. stem is the least, with 2,822 DEGs and 45.5% up-regulated genes (Figure 5C and Supplementary Table 7). Root vs. stem-group with 8,563 DEGs (Figure 5D and Supplementary Table 8), and the up-regulated genes accounted for 54.1%. The results suggest that there is little difference between leaves and stems, while more difference between leaves and roots. There may be some differences in the types and contents of anti-AD active components in the three tissues. Through differential expression analysis, the differential expression of some genes can also be retrieved. According to the distribution characteristics of tissues with the same active ingredients and gene expression, we consider that there is a certain correlation between metabolites and genes. The results showed that there were many differentially expressed genes in the leaves and roots of *V. officinalis*, suggesting that the active components in the leaves were significantly different from those in the roots.

**FIGURE 6:**
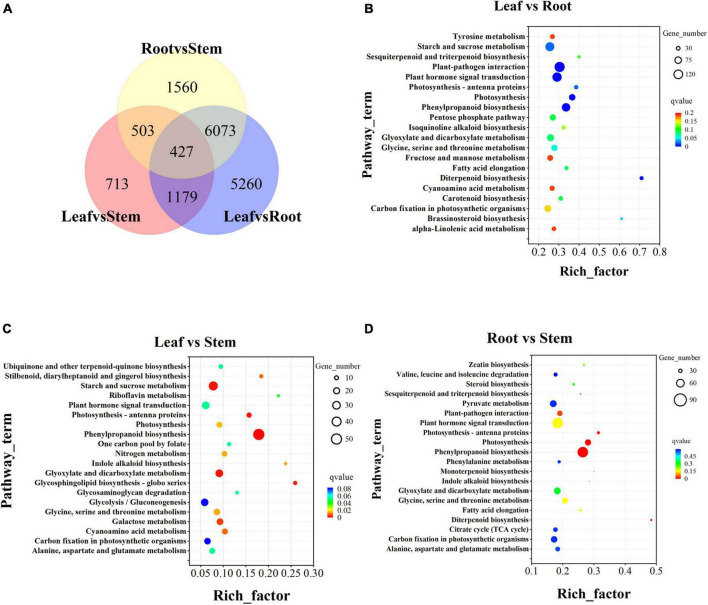
Transcriptome difference analysis. **(A)** Differential venn diagram of three tissues and differential venn diagram between groups. **(B)** Top 20 of KEGG pathway enrichment of Leaf vs. Root DEGs. **(C)** Top 20 of KEGG pathway enrichment of Leaf vs. Stem DEGs. **(D)** Top 20 of KEGG pathway enrichment of Root vs. Stem DEGs. The screening criteria for DEGs are *p* < 0.05 and | log_2_FoldChange| > 1 by DESeq2.

A total of 37,906 unigenes were expressed in the root, stem, and leaf. And 42,313 unigenes were expressed in only one tissue (Supplementary Figure 3). In the comparison of differences between groups, there were 427 DEGs in the three groups of leaf vs. stem, leaf vs. root, and root vs. stem (Figure 6A). To explore the functions and possible regulatory mechanisms of these DEGs, we classified the differential genes and mapped them to the database. The results showed that there were 2,812 DEGs in the leaf vs. root group, which were mapped in 120 KEGG pathways (Figure 6B). The top five pathways were plant pathway interaction (137), Plant hormone signal transmission (114), start and cross metabolism (108), phynopropanoid biosynthesis (94), and Glyoxylate and dicarboxylate metabolism (68). In the KEGG database, 519 DEGs of leaf vs. stem were mapped in 96 pathways (Figure 6C), mainly concentrated in the pathways such as Phylpropanoid biosynthesis (50), Start and sucrose metabolism (33), Glycoxylate and dicarboxylate metabolism (24), Plant hormone signal transmission (24), and Glycolysis/gluconeogenesis (23). The 2,010 DEGs of root vs. stem-group are mapped in 120 pathways (Figure 6D) and mainly reflected in Plant pathway interaction (86), Phenotropic biosynthesis (74), Plant hormone signal transmission (72), Glyoxylate and dicarboxylate metabolism (48), and Pyruvate metabolism (48).

### Content determination results

**FIGURE 7:**
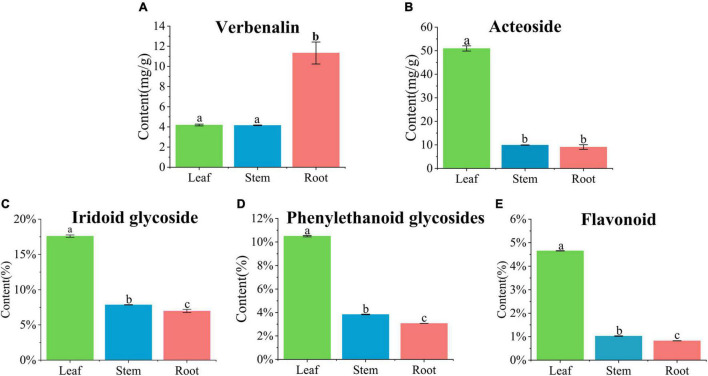
Quantification in the leaf, stem, and root of *Verbena officinalis*. **(A)** Verbenalin. **(B)** Acteoside. **(C)** Total iridoid glycosides. **(D)** Total phenylethanol glycosides. **(E)** Total flavonoids. Values are expressed as the means ± standard errors of three independent samples. Significant differences (*p* < 0.05) were analyzed using Origin2021 and indicated by lowercase letters a, b, and c in the leaf, stem, and root.

The accumulation of iridoid glycosides, phenylethanoid glycosides, and flavonoids in different tissues of *V. officinalis* was studied by content determination. We determined the contents of verbenalin and acteoside by UPLC. From the result, we can know that the contents of the leaf, stem, and root are 4.19 ± 0.09, 4.16 ± 0.03, and 11.33 ± 1.09, respectively (Figure 7A). However, there are some differences in the content. The content of leaf and stem is similar, and roots are significantly up-regulated. The contents of acteoside in leaf, stem, and root were 50.94 ± 1.11, 9.92 ± 0.08, and 9.08 ± 0.97 mg/g, respectively (Figure 7B). The contents of the stem and root were similar and the leaf was significantly up-regulated, which was the same as our understanding that the upper part was an effective component, but verbenalin was not so.

Therefore, the contents of total iridoid glycosides, total phenylethanoid glycosides, and total flavonoids in *V. officinalis* were determined by ultraviolet spectrophotometry. The contents of total iridoid glycosides in the leaves, stems and roots were 17.60, 7.86, and 7.00% (Figure 7C), phenylethanoid glycosides were 10.50, 3.84, and 3.06% (Figure 7D), and flavonoids were 4.65, 1.02, and 0.82% respectively (Figure 7E). The common feature is leaf > stem > root. The results suggest that the genes related to the biosynthetic pathway of the above compounds in *V. officinalis* are highly expressed in the leaf.

### Transcriptome analysis of compounds

The biosynthesis pathway of iridoids mainly involves Terpenoid backbone biosynthesis, Sesquiterpenoid and triterpenoid biosynthesis, Indole alkaloid biosynthesis, Isoquinoline alkaloid biosynthesis, Tropane, piperidine, and pyridine alkaloid biosynthesis. The main pathways involved in the biosynthesis of phenylethanoid glycosides are Tyrosine metabolism, Phenylalanine metabolism, Tryptophan metabolism, and Phenylpropanoid biosynthesis. The main pathways involved in flavonoids biosynthesis are Phenylalanine metabolism, Phenylpropanoid biosynthesis, Flavone and flavonol biosynthesis, and Flavonoid biosynthesis. Annotations of the related pathway genes are shown in Table 3.

**TABLE 3:** Pathways involved in biosynthesis of iridoid glycosides, flavonoids and phenylethanol glycosides.

Pathway name	KO ID	Input number	DEGs number	Leaf vs. Stem	Leaf vs. Root	Stem vs. Root
Tyrosine metabolism	ko00350	127	39	5	34	19
Phenylalanine metabolism	ko00360	122	61	9	26	23
Tryptophan metabolism	ko00380	104	21	3	12	13
Terpenoid backbone biosynthesis	ko00900	153	30	9	28	17
Indole alkaloid biosynthesis	ko00901	30	15	5	9	6
Monoterpenoid biosynthesis	ko00902	22	9	–	8	6
Sesquiterpenoid and triterpenoid biosynthesis	ko00909	37	17	2	14	9
Phenylpropanoid biosynthesis	ko00940	282	127	50	94	74
Flavone and flavonol biosynthesis	ko00944	4	–	–	–	–
Flavonoid biosynthesis	ko00941	60	16	4	12	10
Isoquinoline alkaloid biosynthesis	ko00950	68	24	–	22	12
Tropane, piperidine and pyridine alkaloid biosynthesis	ko00960	53	14	–	13	10

#### Biosynthesis of iridoid glycosides

**FIGURE 8:**
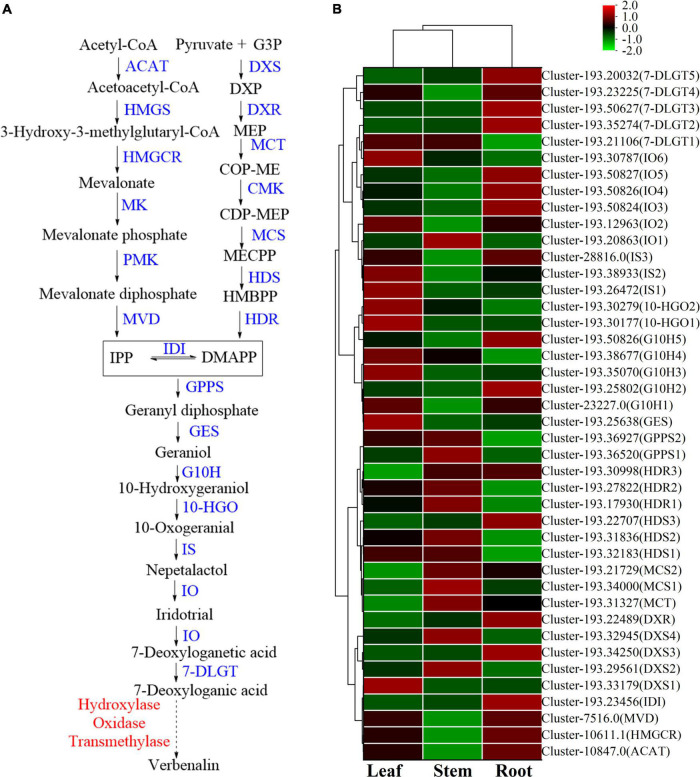
The iridoid glycosides biosynthesis pathway and the heatmap of corresponding genes in *Verbena officinalis*. **(A)** Predicted biosynthetic pathways of iridoid glycosides. Compounds are in black, enzyme names are in blue, and the red have not been reported. The solid arrows are the processes that have been reported, and the dashed lines have not been reported. **(B)** Heatmap of iridoid glycosides pathway DEGs.

There are differences in the expression of genes related to iridoid glycoside synthesis in the root, stem, and leaf of *V. officinalis*. Iridoid glycosides are characteristic compounds in *V. officinalis*. They are special monoterpenoids. Their biosynthesis pathway is the same as that of other terpenoids (Figure 8A). Through transcriptome analysis, 206 unigenes involving the above pathways were annotated, including 61 DEGs. The heat map was drawn based on the FPKM value of the differential genes (Figure 8B and Supplementary Table 9).

Modern studies have shown that the important precursors are IPP and DMAPP. These two substances have two recognized production pathways. One is to take acetyl CoA as the material (Tholl, 2015; Bergman et al., 2019), through acetyl CoA acetyltransferase (ACAT), the MVA pathway of HMG CoA synthase (HMGS), HMG CoA reductase (HMGCR), MVA kinase (MK), phosphomevalonate kinase (PMK), and diphosphomevalonate decarboxylase decarboxylase (MVD). Through database annotation and sequence comparison, we annotated 48 MVA pathway genes in *V. officinalis* transcriptome. There were *ACAT* (cluster-10847.0), *HMGCR* (cluster-10611.1) and MVD (cluster-7516.0) were DEGs and all of them up-regulated in roots. The results suggest that the biosynthesis pathway of terpenoids in *V. officinalis* root may be through the MVA pathway.

The other is the MEP pathway (Volke et al., 2019). The materials are pyruvate and G3P. The enzymes include 1-deoxy-D-xylulose-5-phosphate synthase (DXS), 1-deoxy-D-xylulose-5-phosphate reductoisomerase (DXR), 2-C-methyl-D-erythrol 4-phosphate cydylyl transfer (CMS), 4-diphosphodyl-2-C-methyl-d-phosphate kinase (CMK), 2-C-methyl-d-erythritol 2,4-cyclodiphosphate synthase (MCS), (E)-4-hydroxy-3-methylbut-2-enyl-diphosphate synthase (HDS), and 4-hydroxy-3-methylbut-2-en-1-yldiphosphate reductase (HDR). In recent years, more and more studies tend to believe that the precursors IPP and DMAPP of terpenoid synthesis are mostly provided by the MEP pathway (Ma et al., 2017; Zhu et al., 2022). In this study, 39 single genes were annotated in the MEP pathway, including 14 DEGs. When analyzing the differential expression of genes, we found that *DXS, DXR, MCT, MCS, HDS*, and *HDR* in the MEP pathway are highly expressed in the leaf, suggesting that the synthesis of terpenoids in the leaf is high and realized through MEP pathway.

In the downstream pathway, enzyme genes are also highly expressed in leaves, such as geranyl diphosphate synthase (GPPS), geranyl diphosphate diphosphate (GES), geraniol-10-hydroxylase (G10H), 10-hydroxygeranioxido reduction (10-HGO), iridoid synthesis (IS), and so on. Isomerization between IPP and DMAPP is realized by isopentenyl diphosphate delta isomerase (IDI). *GPPS* is the key enzyme in the formation of GPP from IPP and DMAPP. Simkin et al. identified the enzyme *CrGES* that catalyzes geranyl diphosphate to produce geraniol from *C. roseus* for the first time (Simkin et al., 2013). It should be noted that only two *GES* genes were annotated in the transcriptome, of which cluster-193.45926 had a low expression in all three tissues (FPKM < 1), and another *GES* (cluster-193.25638) had a high expression in leaves. Colu et al. identified *G10H* as *CYP76B6* in *C. roseus*. This enzyme can catalyze geraniol to produce 10-hydroxygeraniol (Collu et al., 2001). Then, 10-hydroxygeraniol is catalyzed by *10-HGO* to generate 10-oxogeranial. There are three genes annotated in which the expression of cluster-193.33255 is low. The other two genes *10-HGO1* (cluster-193.30177) and *10-HGO2* (cluster-193.30279) are differential genes, which are highly expressed in leaf. Therefore, *GES, 10-HGO1* and *10-HGO2*. Then *IS*, iridoid oxidation (IO), 7-deoxyloganic acid glucosyl transferase (7-DLGT), a series of key enzymes catalyze the formation of 7-deoxyglycic acid, which may then be hydroxylated, methylated, and oxidized to produce iridoid glycosides (Sun et al., 2012; Krithika et al., 2015; Ji et al., 2017; Ye et al., 2019).

The genes *IO6* (cluster-193.30787), *HDS2* (cluster-193.31836), and *HDR3* (cluster-193.30998) have the highest FPKM values in this pathway, and they are significantly up-regulated in leaf. Generally speaking, the number of genes with high expression in roots is relatively small, while the expression of related genes in stems is generally low, suggesting that the content of related iridoids glycoside is high in the leaf of plants. Thus, *GES, 10-HGO1*, and *10-HGO2* may be the key enzymes in this process.

Iridoid glycosides are characteristic compounds in *V. officinalis*, mainly including verbenalin, hastatoside, gentiopicroside, aucubin, *etc*., in *V. officinalis* tissue. The total iridoid glycosides in the leaf were significantly increased, but the content of verbenalin in the root was the highest. Five *7-DLGT* DEGs were identified in *V. officinalis* transcriptome, of which cluster-193.35274, cluster-193.50627, cluster-193.23225, and cluster-193.20032 were significantly up-regulated in roots. Meanwhile, the *IO* candidate genes cluster-193.12963, cluster-193.50824, cluster-193.50826, and cluster-193.50827 also showed significant up-regulation in the root. Therefore, the higher expression of these genes in roots may be related to the tissue distribution of verbenalin. The difference in tissue distribution between verbenalin and other iridoid glycosides may be due to the branching of verbenalin in the biosynthetic pathway, and the highly expressed genes *7-DLGT* and *IO* in the root may play a role in the biosynthesis of verbenalin.

#### Biosynthesis of phenylethanoid glycosides

**FIGURE 9:**
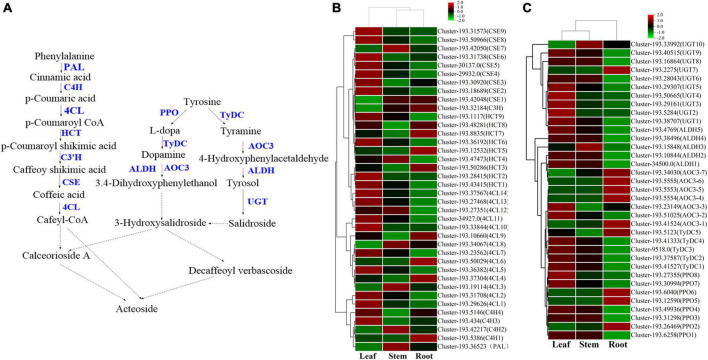
The phenylethanoid glycosides biosynthesis pathway and the heatmap of corresponding genes in *Verbena officinalis*. **(A)** Predicted biosynthetic pathways of phenylethanoid glycosides. Compounds are in black, enzyme names are in blue. The solid arrows are the processes that have been reported, and the dashed lines have not been reported. **(B)** Heatmap of phenylalanine metabolic pathway DEGs. **(C)** Heatmap of dopamine/tyramine pathway DEGs.

The content of phenylethanoid glycosides in *V. officinalis* is high. Acteoside is a typical representative of phenylethanoid glycosides. Its structure is composed of caffeic acid hydroxytyrosol, glucose, and C3-modified rhamnose. The biosynthesis of acteoside (Saimaru and Orihara, 2010) mainly includes the phenylalanine metabolic pathway, dopamine pathway/tyramine pathway, and the glycosylation pathway (Figure 9A). In the *V. officinalis* transcriptome database, the KEGG pathways mainly include phenylpropanoid biosynthesis and tyrosine metabolism. In the above synthetic pathways, 229 related genes were annotated, including 73 DEGs (Figures 9B,C and Supplementary Table 10). The results of the UPLC-MS analysis showed that the phenylethanoid glycosides in *V. officinalis* mainly included acteoside, isoacteoside, and 2’-acetylacetoside.

On the one hand, the phenylalanine metabolic pathway (Zhang et al., 2019; Yang et al., 2021) takes phenylalanine as raw material and generates cafeyl CoA through phenylalanine ammonia lyase (PAL), *trans* cinnate 4-hydroxylase (C4H), 4-coumarate CoA ligase (4CL), shikimate o-hydroxy cinnamoyl transferase (HCT), coumarate-3-hydroxylase (C3H), and caffeoyl shikimate esterase (CSE). In *V. officinalis* transcriptome, the *PAL* (10), *C4H* (10), *4CL* (44), *HCT* (22), *C3H* (3), and *CSE* (18) were all annotated. Most of the candidate genes of *4CL, HCT*, and *CSE* were significantly up-regulated in leaves. On the other hand, in the dopamine pathway/tyramine pathway, tyrosine is catalyzed by polyphenol oxidase (PPO), tyrosine decarboxylase (TyDC), primary amine oxidase (AOC3), and aldehyde dehydrogenase (ALDH) to produce 3.4-dihydroxyphenylthanol. It is connected with a molecule of glucose to produce 3-hydroxysalidroside. Meanwhile, tyrosine can also be catalyzed by *TyDC, AOC3, ALDH*, and *UGT* to produce salidroside. Then 3-hydroxysalidroside is formed by hydroxylation. All the above genes are annotated in the transcriptome, *PPO, TyDC*, and *UGT* were significantly up-regulated in the leaf. So far, the synthetic precursors of acteoside, cafeyl CoA, and 3-hydroxysalidroside have been generated, and then the final product is generated through glycosyl modification and cross-binding reaction, but the downstream process is not clear and needs to be further explored (Alipieva et al., 2014; Zhou et al., 2020).

By referring to the existing research and KEGG pathway, the biosynthetic pathway of acteoside in *V. officinalis* was speculated, and the genes of the phenylalanine metabolic pathway and dopamine pathway/tyramine pathway were all annotated. Therefore, this biosynthetic pathway is feasible in *V. officinalis*. In addition, the genes with the highest expression in this pathway are *CSE* (cluster-193.31738) and *PPO* (cluster-193.31298), which were significantly up-regulated in the leaf, which was consistent with the content determination results.

#### Biosynthesis of flavonoids

**FIGURE 10:**
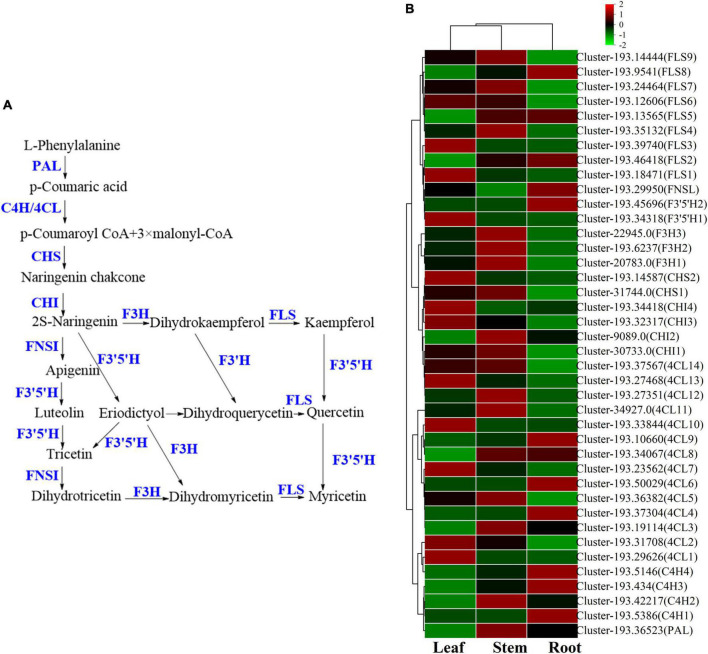
The flavonoids biosynthesis pathway and the heatmap of corresponding genes in *Verbena officinalis*. **(A)** Predicted biosynthetic pathways of flavonoids. Compounds are in black, while enzyme names are in blue. **(B)** Heatmap of flavonoids pathway DEGs.

Flavonoids are common compounds in medicinal plants and one of the main chemical components in *V. officinalis*. Its biosynthetic pathway is relatively clear (Figure 10A). KEGG pathway involved in flavonoid biosynthesis pathway and mainly includes Phylpropanoid biosynthesis, Flavone and flavonol biosynthesis, Flavonoid biosynthesis, *etc*. In the above synthetic pathways, 115 related genes were annotated, including 35 DEGs (Figure 10B and Supplementary Table 11), mainly concentrated in *4CL* (14), *FLS* (9). *V. officinalis* contains many flavonoids.

As the raw material for the synthesis of flavonoids, L-phenylalanine through *PAL, C4H, 4CL*, chalcone synthase (CHS), chalcone isomerase (CHI). A series of enzymes catalyze the formation of flavonoid synthetic skeleton 2*S*-naringeni. In addition, 7 *CHS* and 17 *CHI* genes were annotated, and *CHI* included 4 DEGs. Then 2*S*-naringeni modified by flavone synthase (FNSI), flavanone-3-hydroxylase/naringenin 3-dioxygenase (F3H), Flavonol Synthase (FLS), flavanoid 3’,5’-hydroxylase (F3’5’H) and other enzymes to form myricetin, dihydromyricetin, tricetin and other flavonoids (Zhao et al., 2020; Liu et al., 2021; Lou et al., 2021). All the above genes were annotated in *V. officinalis* transcriptome, including *FNSI* (2), *F3H* (3), *FLS* (14), *F3*’*5*’*H* (8), and *FNSI* (1). In addition, *FLS* (9) and *F3*’*5*’*H* (2) were DEGs.

The results of the UPLC-MS analysis show that it mainly includes acacetin-7-*O*-rutinoside, apigenin-7-*O*-glucoside, *etc*. In the determination of total flavonoids, it was the highest in the leaf. Through KEGG pathway analysis, we mapped the flavonoid biosynthesis pathway. The number of related genes with high expression in the root is relatively small, while more in the leaf. The candidate genes of *4CL, CHI, CHS, F3*’*5*’*H, FLS*, and other enzymes are significantly up-regulated in the leaf. Among them, *F3*’*5*’*H* has 8 candidate genes were annotated, and only *F3*’*5*’*H1* (cluster-193.34318) was significantly up-regulated in leaf, which was consistent with the content determination results. At the same time, it also suggested that these genes may be the rate-limiting enzyme of flavonoids in *V. officinalis*.

### Transcription factors analysis

Transcription factors (TFs) play a vital role in plant growth. They affect the production and accumulation of plant secondary metabolites by promoting or inhibiting genes expression. A total of 3,717 genes were annotated in *V. officinalis* transcriptome, belonging to 90 categories of TFs (Supplementary Table 12). Among them, the largest number of genes was C2H2 (403, accounting for 12.71%), followed by bHLH (basic helix loop helix, 158, accounting for 4.98%), AP2/ERF ERF (apetala2/ethylene-responsive factor, 152, accounting for 4.79%), and C3H (cys3his zinc finger domain-containing protein, 131, accounting for 4.13%).

**FIGURE 11:**
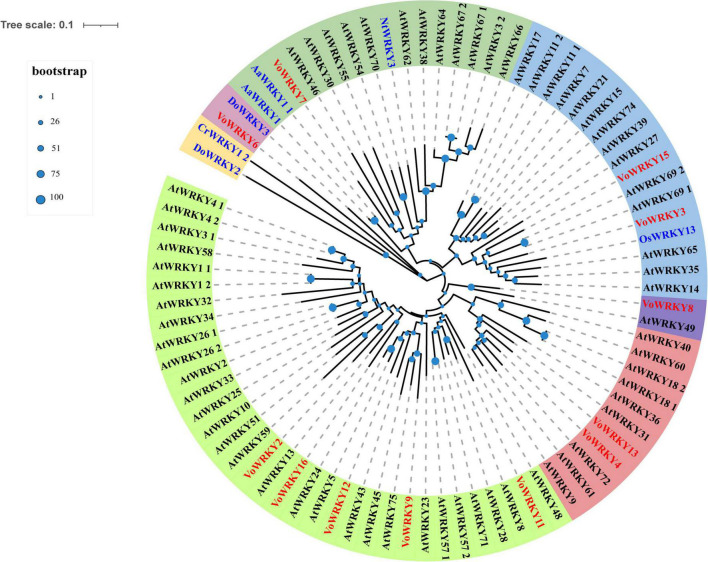
Phylogenetic tree of WRKY transcription factors in *Verbena officinalis.* Red font is *Verbena officinalis* TFs, black font is *Arabidopsis thaliana* transcription factor, and blue font is transcription factor of other species. The size of the blue node represents the bootstrap value.

There are 111 VoWRKYs were annotated in *V. officinalis* transcriptome, accounting for 3.50% of the total transcription factor genes. To explore the role of WRKYs in the growth and development of *V. officinalis*, 16 gene sequences with |log_2_FoldChange| > 4 in *V. officinalis* WRKY were screened (figure, the expression statistics of genes with |log_2_FoldChange| > 4 in *V. officinalis* WRKY). After removing redundant genes, 12 genes (Supplementary Table 13) were selected to build a phylogenetic tree with AtWRKYs, NtWRKY, DoWRKYs, CrWRKY, OsWRKY, and AaWRKYs (Figure 11). According to the results of phylogenetic analysis, VoWRKY6 and DoWRKY3 are in the same branch, and their gene sequences are similar. Therefore, VoWRKY6 may have similar functions to DoWRKY3, affect TPS, and regulate the biosynthesis of terpenoids in *V. officinalis*. VoWRKY7 is close to AaWRKY1 1, which may also play a role in regulating terpene biosynthesis. OsWRKY13 and AtWRKY23 are related to flavonoid biosynthesis. Therefore, VoWRKY3, VoWRKY9, and VoWRKY12, which are close to them, may also play a role in regulating the flavonoid biosynthesis of *V. officinalis*.

## Discussion

*Verbena officinalis* is a medicinal plant of the genus *Verbena*. In addition, its relatives, *Verbena bonariensis* L., *Verbena brasiliensis* Vell, *Verbena hybrid* Voss, *Verbena tenera* Sprang, *Verbena hastata* L., *Verbena litoralis* Kunth, *etc*. also belong to the genus of *Verbena*, and most of which have good medicinal values and are used as folk medicine. To explore the genetic relationship between *Verbena* plants, Yuan et al. used the pentatricopeptide repeat gene family to analyze the phylogeny of *Verbena* plants. Hence, the phylogenetic framework was constructed, and it provided an important basis for the phylogenetic relationship of *Verbena* plants (Yuan et al., 2010). Ruzicka et al. constructed the phylogenetic tree of *Verbena* by comparing the internal transcribed spacer sequences and molecular markers of *Verbena*. Under the two conditions, the difference between *V. officinalis* and *V. hastata* was the smallest (Ruzicka et al., 2009).

Based on the genetic relationship and genetic similarity, *Verbena* plants may have similar secondary metabolites and pharmacological effects (Gong et al., 2022). De Lima et al. analyzed *V. littoralis* by UHPLC-ESI-HRMS and found that phenylethanoid glycosides, iridoid glycosides, and triterpenoids were identified in plants (De Lima et al., 2018). Soares et al. (2020) analyzed the main chemical components of *V. minutiflora* by HPLC-DAD. The results showed that iridoid glycosides, phenylethanoid glycosides, and flavonoids all had high contents in plants. el-Hela et al. (2000) isolated the chemical constituents of *V. bipinnatifida*, and successfully identified two iridoid glycosides and two phenylethanoid glycosides. A large number of literature studies have shown that iridoid glycosides and phenylethanoid glycosides are common in *Verbena* plants, and verbenalin, hastatoside, acteoside, and isacteoside are high content in these plants. This also suggests that these plants may have similar pharmacological activities.

At present, there are few studies on the *Verbena* plants. Only *V. officinalis* and *V. bonariensis* have some chemical constituents and pharmacological effects research. In previous reports, nuclear magnetic resonance (Shu et al., 2014; Luo et al., 2021), HPLC-DAD (Liu et al., 2012), HPLC-MS (El-Hela et al., 2010) and other technologies were used to study the chemical components and content of *V. officinalis*. In this study, we used UPLC-MS technology to identify the chemical components in *V. officinalis* extract. We not only used the chromatographic separation technology, but also combined the high selectivity and strong structural identification ability of mass spectrometry. A total of 16 compounds in *V. officinalis* extract were detected by this method, and the structure of each compound was further confirmed by multi-stage full scan mass spectrometry. Iridoid glycosides, phenylethanoid glycosides, and flavonoids, as the main components of *V. officinalis*, are considered the main components for anti-AD effect. Therefore, we preliminarily investigated the activity of the compounds with the help of molecular docking technology. The iridoid glycosides, phenylethanoid glycosides, and flavonoids of *V. officinalis* showed good activity in the anti-AD molecular docking experiment. Iridoid glycosides showed good binding to inflammation-related proteins NLRP3, and phenylethanoid glycosides showed good binding to NLRP3 and BACE1. Flavonoids have a good binding effect with target proteins NLRP3, BACE1, AChE, and GSK-3β. This result suggested that the compounds in *V. officinalis* had a good potential for anti-AD activity.

To further explore the active components against AD, we divided *V. officinalis* tissue into three ones: leaf, stem, and root. The contents of verbenalin and acteoside were determined by UPLC. The results showed significant differences in the three tissues. However, the content of verbenalin is the highest in the root, which is contrary to the fact that the upper part is used as medicine. Therefore, we determined the tissue content differences of total iridoid glycosides, total phenylethanoid glycosides, and total flavonoids by a UV spectrophotometer. The three components have the same tissue distribution characteristics. The content is leaf > stem > root.

Meanwhile, we obtained the transcriptome date by RNA-Seq and annotated the gene functions through database comparison. In addition, through KEGG pathway enrichment and literature retrieval, we speculated and mapped the biosynthetic pathways of iridoid glycosides, phenylethanoid glycosides, and flavonoids. The related genes and DEGs of pathways were identified, and the key rate-limiting enzymes of iridoid glycosides may be *GES, 10-HGO1* and *10-HGO2*. In addition, the tissue distribution of verbenalin is special, which may be related to the branching of the biosynthetic pathway in the downstream approach. The accumulation of metabolites in different tissues is not completely consistent with the level of gene expression, indicating that the genes related to substance synthesis and decomposition are differentially controlled or regulated. The speculation and gene identification of the phenylethanoid glycoside biosynthesis pathway have been predicted, but the search for key rate-limiting enzymes needs further experiments. *F3*’*5*’*H1* was speculated to be the key gene of flavonoid biosynthesis because of its unique up-regulation in candidate genes. TFs analysis also led us to speculate that the WRKY related to the biosynthesis of iridoid glycosides in transcriptome may be VoWRKY6 and VoWRKY7, while VoWRKY3, VoWRKY9, and VoWRKY12 may be involved in regulating the biosynthesis of flavonoids in *V. officinalis*.

However, our experiment has some shortcomings. There are many key enzymes involved in the biosynthetic pathways, and we have not verified their functions. In addition, the molecular docking technology verified that the anti-AD activity components of the compounds remained at the virtual level. Therefore, our next step is to verify the function of key enzymes involved in the pathway. At the same time, we also plan to improve the exploration of anti-AD activity to the pharmacological level, and establish an AD model through model organism *Caenorhabditis elegans* to verify the activity of compounds.

Previously, there was no report on the analysis of *V. officinalis* transcriptome. Therefore, we creatively analyzed the biosynthesis pathway of iridoid glycosides, phenylethanoid glycosides, and flavonoids by using RNA-Seq technology, and combinedthe results with content determination to explore the biosynthetic pathway of anti-AD ingredients. First, UPLC-MS helped us to identify the main chemical constituents of *V. officinalis*. After confirming the existence of chemical components, we investigated the activities of these compounds by molecular docking technology and directly explored the anti-AD activity of the compounds in *V. officinalis*. This method of combining chemical analysis and transcriptome analysis is novel and has great application potential in the future. Through this technique, we investigated the possible regulatory mechanism of the active components in *V. officinalis*. Some compounds in *V. officinalis* show predominant multi-target anti-AD activity, so they can further target the pathogenesis. Natural products have significant advantages in finding new anti-AD agents and provide clues for the prevention and treatment of AD. Meanwhile, because *V. officinalis* has similar active components and gene sequences with *Verbena* plants, it is suggested that other *Verbena* plants may have similar pharmacological activities with *V. officinalis*. At the same time, the study of anti-AD active components in *V. officinalis* will provide a reference for the research and development of other *Verbena* plants.

## Data availability statement

The datasets presented in this study can be found in online repositories. The names of the repository/repositories and accession number(s) can be found below: https://www.ncbi.nlm.nih.gov/, PRJNA842648.

## Author contributions

CW, XY, and ZL conceived and designed the project. SP, KY, and FL performed the experiments, processed the experimental data and analyzed it. SP wrote the manuscript. HY helped JG draw the figures in the article. CW, GL, and HL checked and corrected the manuscript. All authors read and approved the final manuscript.

## Footnotes

1
https://pubchem.ncbi.nlm.nih.gov/

2
https://www.rcsb.org/

3
https://itol.embl.de/
