# Supplementary material for: Integrating transcriptome and chemical analyses to reveal the anti-Alzheimer’s disease components in Verbena officinalis Linn
Source: Front Plant Sci. 2022 Aug 4;13:955075. doi: 10.3389/fpls.2022.955075 (PMC9386363; doi:10.3389/fpls.2022.955075)
Supplement: Supplementary file 1 [file Data_Sheet_1.DOCX]

Notes on Supplementary materials

1. **Supplementary Figure 1**


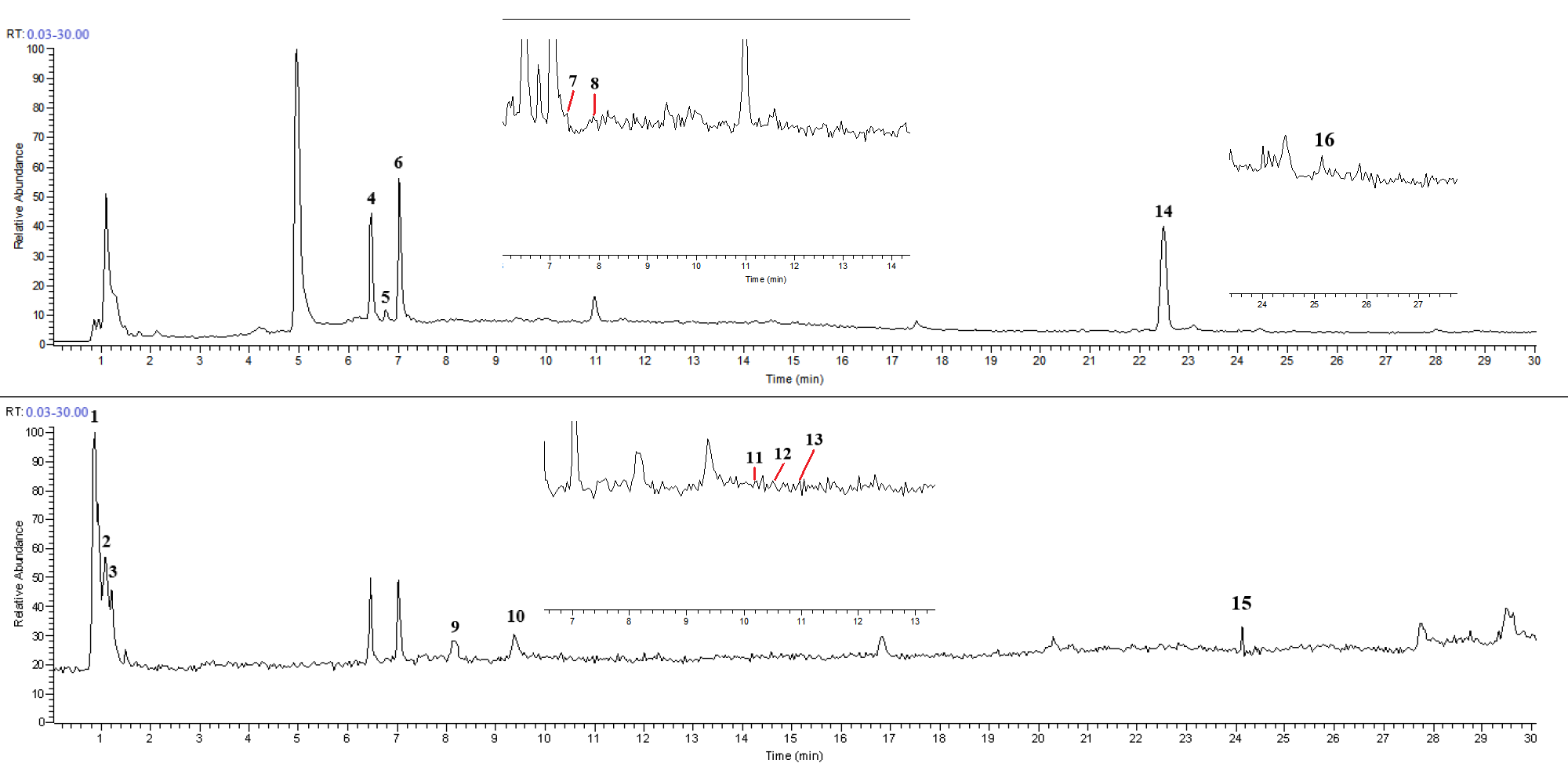


**Supplementary Figure 1.** The top is a scan in positive ion mode, the bottom is a scan in negative ion mode of *V. officinalis.* The numbers indicate the peaks of compounds: 1 2'-Acetylacetoside, 2 Aucubin, 3Swertiamarine, 4 Hastatoside, 5 Gentiopicroside, 6 Verbenalin, 7 Apigenin-7-O-glucoside, 8 3,4-DihydroVerbenalin, 9 Glucosyl-6-pedalitin, 10 Acetoside, 11 Cistanoside F, 12 Jionoside D, 13 Isoacteoside, 14 4'-Hydroxyl wogonoside, 15 Acacetin, 16 Acacetin-7-O-rutinoside.

1. **Supplementary Figure 2**

**
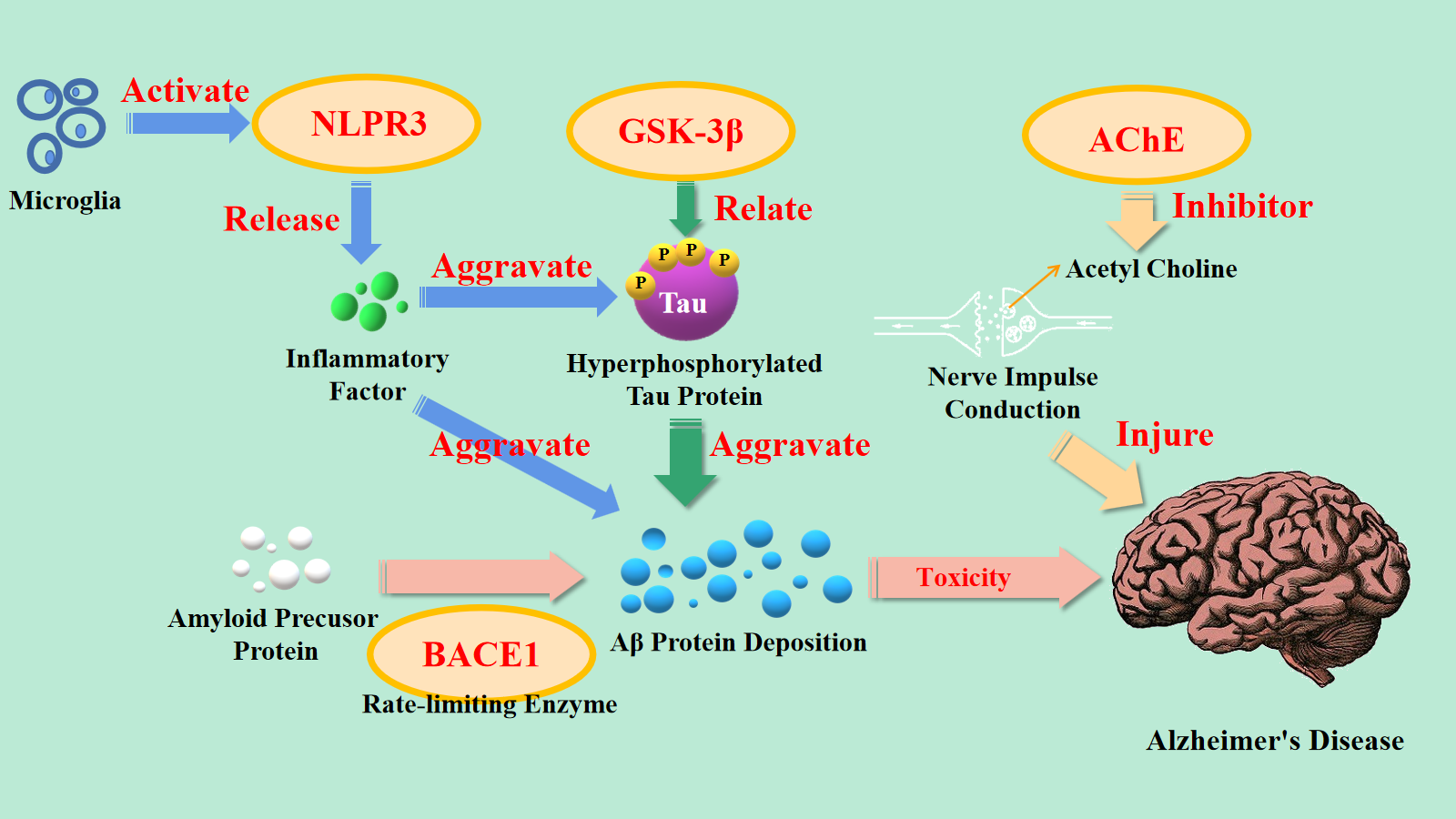
**

**Supplementary Figure 2.** Mechanisms of anti-AD target proteins in molecular docking studies.

1. **Supplementary Figure 3**


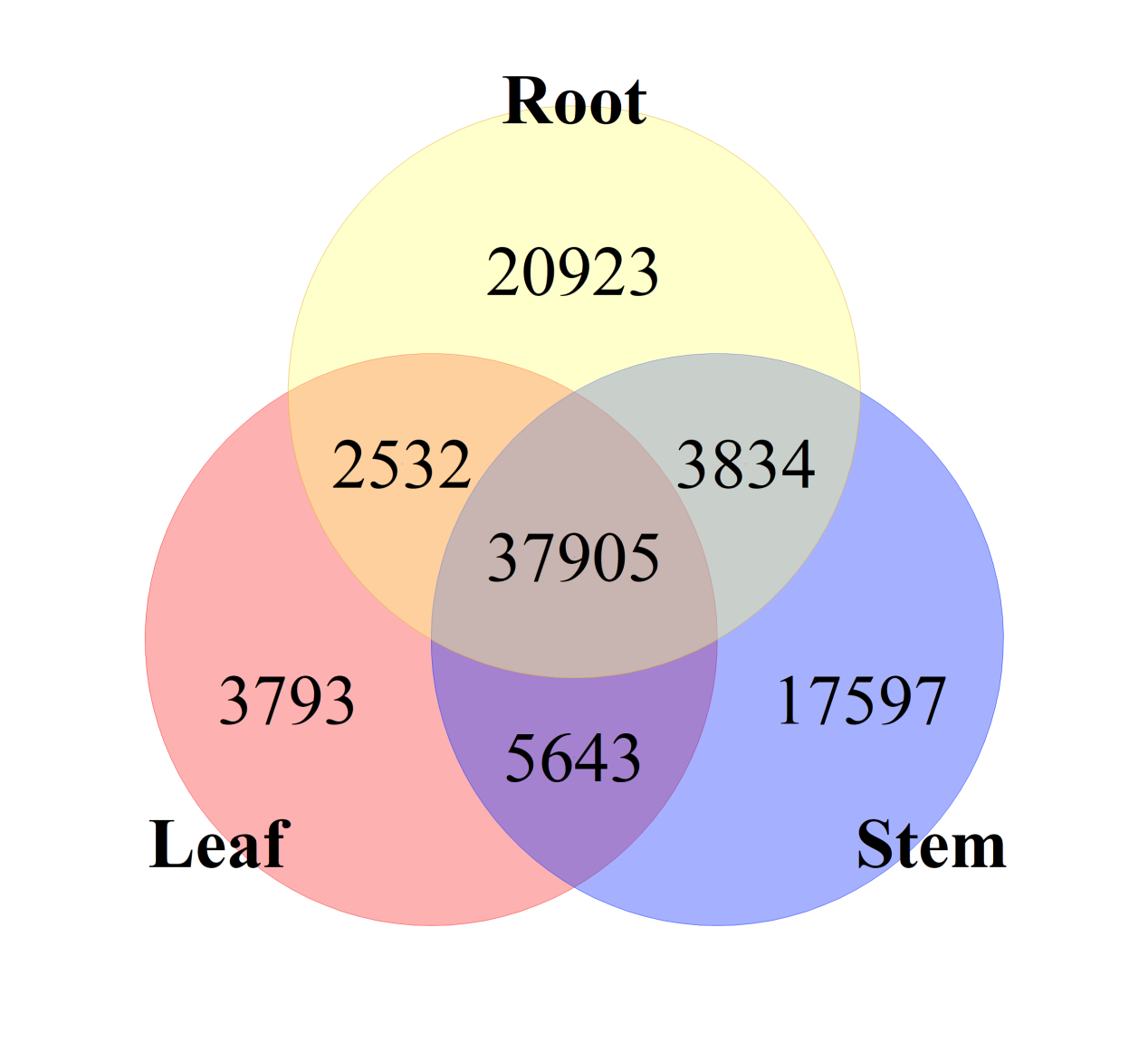


**Supplementary Figure 3.** Venn diagram of three tissue differential genes.

1. **Supplementary Table**

**Supplementary Table 1.** Summary of sample sequencing data quality.

**Supplementary Table 2.** Length frequency distribution of the spliced transcripts and genes.

**Supplementary Table 3.** Classification results of KOG enrichment.

**Supplementary Table 4.** Classification results of KEGG enrichment.

**Supplementary Table 5.** Classification results of GO database enrichment.

**Supplementary Table 6.** DEGs of Leaf vs. Root group.

**Supplementary Table 7.** DEGs of Leaf vs. Stem group.

**Supplementary Table 8.** DEGs of Root vs. Stem group.

**Supplementary Table 9.** Iridoid glycoside biosynthesis genes identified transcriptome.

**Supplementary Table 10.** Phenylethanoid glycosides biosynthesis genes identified transcriptome.

**Supplementary Table 11.** Flavonoids biosynthesis genes identified transcriptome.

**Supplementary Table 12.** TFs annotation result.

**Supplementary Table 13.** WRKY TFs for phylogenetic analysis in *V. officinalis.*
